# Antitumorigenic and antiangiogenic efficacy of apatinib in liver cancer evaluated by multimodality molecular imaging

**DOI:** 10.1038/s12276-019-0274-7

**Published:** 2019-07-08

**Authors:** Qian Liang, Lingxin Kong, Yang Du, Xu Zhu, Jie Tian

**Affiliations:** 1 CAS Key Laboratory of Molecular Imaging, the State Key Laboratory of Management and Control for Complex Systems, Institute of Automation, Chinese Academy of Sciences, 100190 Beijing, China; 2 University of Chinese Academy of Sciences, 100080 Beijing, China; 3 Beijing Key Laboratory of Molecular Imaging, 100190 Beijing, China; 4 Key Laboratory of Carcinogenesis and Translational Research (Ministry of Education/Beijing), Department of Interventional Therapy, Peking University School of Oncology, No. 52 Fucheng Road, Haidian District, 100142 Beijing, China; 5 Beijing Advanced Innovation Center for Big Data-Based Precision Medicine, School of Medicine, Beihang University, 100191 Beijing, China; 6 Engineering Research Center of Molecular and Neuro Imaging of Ministry of Education, School of Life Science and Technology, Xidian University, Xi’an, 710126 Shaanxi, China

**Keywords:** Cancer imaging, Targeted therapies

## Abstract

Hepatocellular carcinoma (HCC) is one of the most common causes of cancer-related mortality worldwide. Sorafenib is the standard first-line treatment for advanced HCC, but its efficacy is limited. Apatinib is a small-molecule tyrosine kinase inhibitor that has shown promising antitumor effects in gastric and non-small cell lung cancers in clinical trials, but there have been only a few studies reporting its anti-HCC effects in vitro and in HCC xenograft models. Hence, our present study systemically investigated and compared the antitumorigenic and antiangiogenic efficacy of apatinib and sorafenib in HCC in vitro and in vivo using multimodality molecular imaging, including bioluminescence imaging (BLI), bioluminescence tomography (BLT), fluorescence molecular imaging (FMI), and computed tomography angiography (CTA). Moreover, the safety and side effects of the two drugs were systemically evaluated. We found that apatinib showed a comparable therapeutic efficacy to sorafenib for the inhibition of HCC. The drug safety evaluation revealed that both of these drugs caused hypertension and mild liver and kidney damage. Sorafenib caused diarrhea, rash, and weight loss in mice, but these effects were not observed in mice treated with apatinib. In conclusion, apatinib has similar antitumorigenic and antiangiogenic efficacy as sorafenib in HCC with less toxicity. These findings may provide preclinical evidence supporting the potential application of apatinib for the treatment of HCC patients.

## Atherosclerosis: halting plaque in its tracks

Researchers have combined different sophisticated imaging techniques to assess the safety and efficacy of liver cancer therapy in animal models. Many hepatocellular carcinoma (HCC) patients respond to sorafenib, but this drug is expensive and may cause severe side-effects. Qian Liang at China’s Institute of Automation, Beijing, and colleagues have employed cutting-edge imaging technologies to study an alternative drug, apatinib, which has shown promise for stomach and lung cancer and has an excellent safety profile. Using bioluminescence imaging, the researchers could directly visualize apatanib-mediated inhibition of tumor growth in live mice much earlier than would be possible with other methods. The researchers subsequently used additional imaging techniques to demonstrate that apatanib inhibits tumor blood vessel growth. These findings reveal a promising alternative treatment for HCC, as well as a powerful strategy for drug testing in animals.

## Introduction

Liver cancer is a highly fatal cancer worldwide and is the second leading cause of cancer death in men and the sixth in women^1^. The disease is often detected at a late stage. Computed tomography (CT) and magnetic resonance imaging (MRI) are used to evaluate the efficacy of clinical drug treatment according to the gold standard response evaluation criteria in solid tumors^2^. This approach is only suitable for monitoring changes in tumor anatomy in advanced hepatocellular carcinoma (HCC) patients. However, delayed assessment can result in patients missing the optimal treatment window, which reduces the 5-year survival rate. As such, more sensitive and effective imaging methods for the early evaluation of drug efficacy are needed in both preclinical and clinical studies.

Molecular imaging allows noninvasive and dynamic assessment of physiological processes at the molecular and cellular levels in intact living subjects, which can reveal biological activity and drug responses, thereby allowing the assessment of drug efficacy at an early therapeutic stage^3,4^. Optical molecular imaging techniques, such as bioluminescence imaging (BLI) and fluorescence molecular imaging (FMI), are essential in cancer detection, drug development, and evaluation of treatment response for their high sensitivity, no radioactivity, ease of use, and relatively low cost^3,5^. However, these methods suffer from the limited penetration depth and the lack of anatomical structure details. Combining optical imaging with CT or MRI could potentially overcome these problems.

Targeted drug therapy is the most common treatment for advanced cases of HCC, and sorafenib is considered the standard first-line treatment for advanced HCC^6^. However, in some patients, it is necessary to reduce the drug dose to avoid side effects such as diarrhea, vomiting, and hand-foot syndrome^7,8^, which limit the antitumor efficacy. Moreover, the high cost of sorafenib makes it untenable for long-term treatment of HCC patients. For this reason, new targeted drugs for HCC are currently being developed and tested. Since HCC is characterized as a highly vascularized tumor, suppressing angiogenesis is an attractive treatment strategy^6,9,10^. Sorafenib can effectively block tumor angiogenesis in an HCC model and targets multiple receptor tyrosine kinases (RTKs), including vascular endothelial growth factor receptor (VEGFR)-2, platelet-derived growth factor receptor (PDGFR), FLT3, Ret, and c-Kit^11^. Similarly, apatinib is a small-molecule tyrosine kinase inhibitor that can inhibit HCC progression through multiple RTKs, including VEGFR-2, PDGFR, IGF-IR, and Dtk^12^. In addition, apatinib has shown promising antitumor effects in gastric and non-small cell lung cancers in clinical trials^10^. Surprisingly, only a few studies reported its anti-HCC effects in vitro and in mouse xenograft models of human HCC^12,13^.

In this study, we systemically investigated and compared the antitumor effects of apatinib and sorafenib on HCC using multimodality molecular imaging approaches. Drug treatment efficacy was assessed in vitro using several human HCC cell lines and in vivo using subcutaneous and orthotopic HCC mouse models. The safety and side effects of these drugs were also systemically evaluated. Our study provides preclinical evidence for the efficacy of apatinib in the treatment of HCC patients.

## Materials and methods

### Materials and reagents

HepG2 and Hep3B HCC cell lines were obtained from the American Type Culture Collection (Manassas, VA, USA). HUH7, SMMC-7721, and BEL-7402 HCC cell lines were obtained from Shanghai Genechem Co. (Shanghai, China). The HepG2-Red-fLuc cell line was purchased from PerkinElmer (Waltham, MA, USA). Dulbecco’s modified Eagle’s medium (DMEM), Roswell Park Memorial Institute (RPMI) 1640 medium, and fetal bovine serum (FBS) were obtained from HyClone (Logan, UT, USA). Apatinib and sorafenib were obtained from Jiangsu Hengrui Medicine Co. (Jiangsu, China) and Bayer (Leverkusen, Germany), respectively. AngioSense 750 EX and d-luciferin were obtained from PerkinElmer. Fenestra VC was from ART Advanced Research Technologies (Montreal, QC, Canada).

### Cell culture

HepG2-Red-fLuc, HUH7-fLuc, and Hep3B cells were cultured in DMEM and SMMC-7721-fLuc, and BEL-7402 cells were cultured in RPMI 1640 medium supplemented with 10% FBS in a controlled environment (37 °C and 5% CO_2_).

### In vitro cell viability assay

We assessed the sensitivity of HCC cells for the treatment of apatinib and sorafenib using HUH7, HepG2, Hep3B, SMMC-7721, and BEL-7402 cell lines. The cell suspension was added to each well of a 96-well plate followed by overnight culture. After refreshing the culture medium with PBS, a series of concentrations of apatinib (0, 1, 10, 20, and 50 μM) and sorafenib (0, 1, 10, 20, and 50 μg/ml) were added. After 24 h, Cell Counting Kit (CCK)-8 solution was added to each well, followed by incubation for 4 h, and the absorbance at 450 nm was measured with a microplate reader (BioTek Instruments, Winooski, VT, USA). The cell survival rate was calculated using the following formula:

$${\mathrm{Cell}}\,{\mathrm{survival}}\,{\mathrm{rate}} = \frac{{{\mathrm{As}} - {\mathrm{Ab}}}}{{{\mathrm{Ac}} - {\mathrm{Ab}}}} \times 100\%$$

where As is the absorbance of cells treated with the drug, Ac is that of control cells treated with phosphate-buffered saline (PBS), and Ab is that of PBS.

For HCC cells expressing the luciferase gene (HepG2-Red-fLuc, SMMC-7721-fLuc, and HUH7-fLuc) grown in 96-well plates, BLI was performed after 24 h of drug application to evaluate the treatment efficacy.

### Animal model

Male athymic Balb/c nude mice (4–5 weeks old) were purchased from Vital River Laboratory Animal Technology Corp. (Beijing, China). Animal experiments were performed according to the guidelines of the Institutional Animal Care and Use Committee of Peking University (permit no. 2011-0039).

The subcutaneous liver tumor model was established in Balb/c nude mice by injecting 1 × 10^6^ HepG2-Red-fLuc or SMMC-7721-fLuc cells into the right lower flank of mice. The orthotopic liver tumor model was generated by inoculating 1 × 10^6^ HepG2-Red-fLuc cells into the liver lobe of mice through laparotomy under isoflurane anesthesia. After the surgery, the peritoneum and skin of the mice were sutured with absorbable surgical lines, and the mice were placed in a warm box until they woke up.

### Drug treatment

After inoculation of liver tumor cells, liver tumor growth was monitored by measuring BLI signal intensity and tumor volume. At 7 days after liver tumor cell implantation, subcutaneous or orthotopic tumor-bearing mice were randomly divided into the control, sorafenib and apatinib treatment groups. Apatinib and sorafenib were dissolved in 1:1:8 DMSO/Cremphor EL/PBS^14^. The mice treated with apatinib were orally gavaged with 70 mg/kg apatinib every 2 days for a total of six times, while the sorafenib group was gavaged with 62 mg/kg sorafenib every 2 days. The control mice were given an equal volume of diluted solvent with sterile water.

### In vivo BLI of subcutaneous and orthotopic HCC models during drug treatment

Before imaging, mice were anesthetized with 2% isoflurane and intraperitoneally injected with d-luciferin (150 mg/kg body weight) for 8 min. BLI was carried out with an IVIS spectrum optical imaging system (PerkinElmer) every 2 days during drug treatment for a total of six times. Changes in the BLI signal intensity in regions of interest at different time points were quantitatively analyzed to evaluate the antitumor efficacy of the drugs.

### Micro-(μ)CT/BLI system

A schematic illustration of the μCT/BLI system is shown in Fig. S1. The μCT and BLI systems share the same imaging platform, and the optical imaging and CT instruments are arranged on a rotating disk. The μCT imaging system consisted of an X-ray source (UltraBright; Oxford Instruments, Concord, MA, USA), an imaging platform with a mouse holder, and a flat panel X-ray detector (C7942CA-02; Hamamatsu Photonics, Hamamatsu, Japan)^15^. The BLI system for capturing bioluminescent signals on the body surface consisted of an electron-multiplying charge-coupled device camera (EMCCD) (iXonEM+888; Andor Technology, Belfast, Ireland) and imaging stage^16^.

### In vivo bioluminescence tomography (BLT) of the orthotopic HCC model during drug treatment

On day 20 post-drug treatment, the three-dimensional (3D) tomographic imaging data and the bioluminescent surface signals were acquired using the μCT/BLI system, and the 3D distribution of bioluminescence of the orthotopic liver tumor in the three groups was reconstructed. Before BLT imaging, the mouse was anesthetized with 2% isoflurane and intraperitoneally injected with d-luciferin for 8 min; it was then fixed on the imaging platform with a respiratory mask connected to the gas anesthesia vaporizer^17^. Images were captured with the EMCCD camera at different angles (0°, 90°, 180°, and 270°) with the following parameters: binning = 1 and exposure time = 10 s. The 3D anatomical data were obtained by μCT scanning. The major organs, including muscles, heart, lung, liver, kidney, and bone, were segmented to generate the heterogeneous mouse model. Moreover, the assembled mouse body was discretized into the surface and volumetric mesh data for BLT reconstruction. 3D BLT reconstruction procedure of the orthotopic liver tumor model is shown in Fig. S2. The detailed BLT reconstruction method is available in the Supplementary Information. The voltage and current of the X-ray tube were set to 80 kV and 75 μA, respectively, and the integration time of the detector was 0.50 s. The parameters of the rotating motor were as follows: rotating speed = 5°/s, angle interval = 1°, and number of projections = 360.

### Imaging of angiogenesis in the orthotopic liver tumor model

To investigate whether apatinib and sorafenib could inhibit tumor angiogenesis in the orthotopic liver tumor model, we carried out FMI of tumor angiography on day 20 post-drug treatment. AngioSense 750 EX, a fluorescent blood pool imaging agent, was injected through the tail vein at a dose of 2 nmol/100 μL per mouse. FMI images of orthotopic tumors were acquired at different time points (pretreatment and 0, 4, 8, 12, 24, and 48 h post-treatment). We also acquired BLI images of the tumors at 48 h. Mice were sacrificed, and liver lobes with tumors were dissected for *ex vivo* optical imaging (white light, BLI, and FMI).

Moreover, on day 20 post-drug treatment, the Fenestra VC contrast agent (10 μL/g) was intravenously injected into the orthotopic HCC tumor-bearing mice approximately 15 min prior to imaging by CT angiography (CTA)^18^. CTA data were processed with Amira v.5.2.1 (Thermo Fisher Scientific, Waltham, MA, USA).

### Body weight and tumor volume measurements

Body weight was measured using an electronic balance every 2 days during treatment. Liver tumor size was measured using digital Vernier calipers, and the tumor volume was calculated using the following formula:

$${\mathrm{Tumor}}\,{\mathrm{volume}} = \frac{{\mathrm{1}}}{{\mathrm{2}}} \times ({\mathrm{tumor}}\,{\mathrm{length}}) \times ({\mathrm{tumor}}\,{\mathrm{width}})^{\mathrm{2}}$$

### Drug toxicity analysis

Mouse urine samples were collected via metabolic cages (Tecniplast, Buguggiate, Italy) after drug treatment for analysis of 24 h urine creatinine and urine protein levels. Liver function was evaluated by monitoring serum alanine transaminase (ALT) and aspartate transaminase (AST) levels after treatment in three groups. The blood pressure of tumor-bearing mice before and after treatment was measured noninvasively with a blood pressure monitor (BP-2010A; Softron Biotechnology, Tokyo, Japan).

### Histological study

At the end of drug treatment, the mice were sacrificed, and the major organs, including the heart, liver, spleen, lung, and kidney, as well as tumors, were removed. The tissue specimens were fixed in 4% formalin and embedded in paraffin, and 5-μm sections were cut and stained with hematoxylin and eosin (H&E). Images were acquired with a light microscope (DMI3000; Leica, Wetzlar, Germany).

### CD31 and Ki67 immunohistochemistry and terminal deoxynucleotidyl transferase dUTP nick end labeling (TUNEL)

We carried out CD31 and Ki67 immunohistochemistry and TUNEL staining of the tumor samples on day 25 post-treatment. Tumor samples were fixed in 4% formalin and embedded in paraffin. The tissue blocks were cut into sections at a thickness of 5 μm that were deparaffinized and rehydrated. Endogenous peroxidase activity was quenched by incubation in 1% H_2_O_2_ for 10 min, and antigen retrieval was performed for 10 min at room temperature. The sections were blocked by incubation in 10% goat serum for 1 h and then incubated overnight at 4 °C with primary antibody against CD31 (cat. no. Ab28364; Abcam, Cambridge, UK) or Ki67 (cat. no. Ab15580; Abcam, Cambridge, UK). The following day, the sections were incubated with secondary antibody at 37 °C for 20 min, and immunoreactivity was visualized with a diaminobenzidine color reagent kit (cat. no. HBT-3032; Hycult Biotech, Wayne, PA, USA). Apoptotic cells were detected with a TUNEL apoptosis detection kit (cat. no. 11684817910; Roche Diagnostics, Basel, Switzerland).

### Statistical analysis

Data are expressed as the mean ± standard error of the mean and were analyzed using Prism 5 software (GraphPad Inc., La Jolla, CA, USA). Differences in mean values of different groups were compared with Student’s *t*-test, with a *P*-value < 0.05 considered statistically significant. Concretely, * indicates *P*-value < 0.05, ** indicates *P*-value < 0.01, and *** indicates *P*-value < 0.001.

## Results

### Effect of apatinib and sorafenib on HCC cell viability

**Fig. 1:**
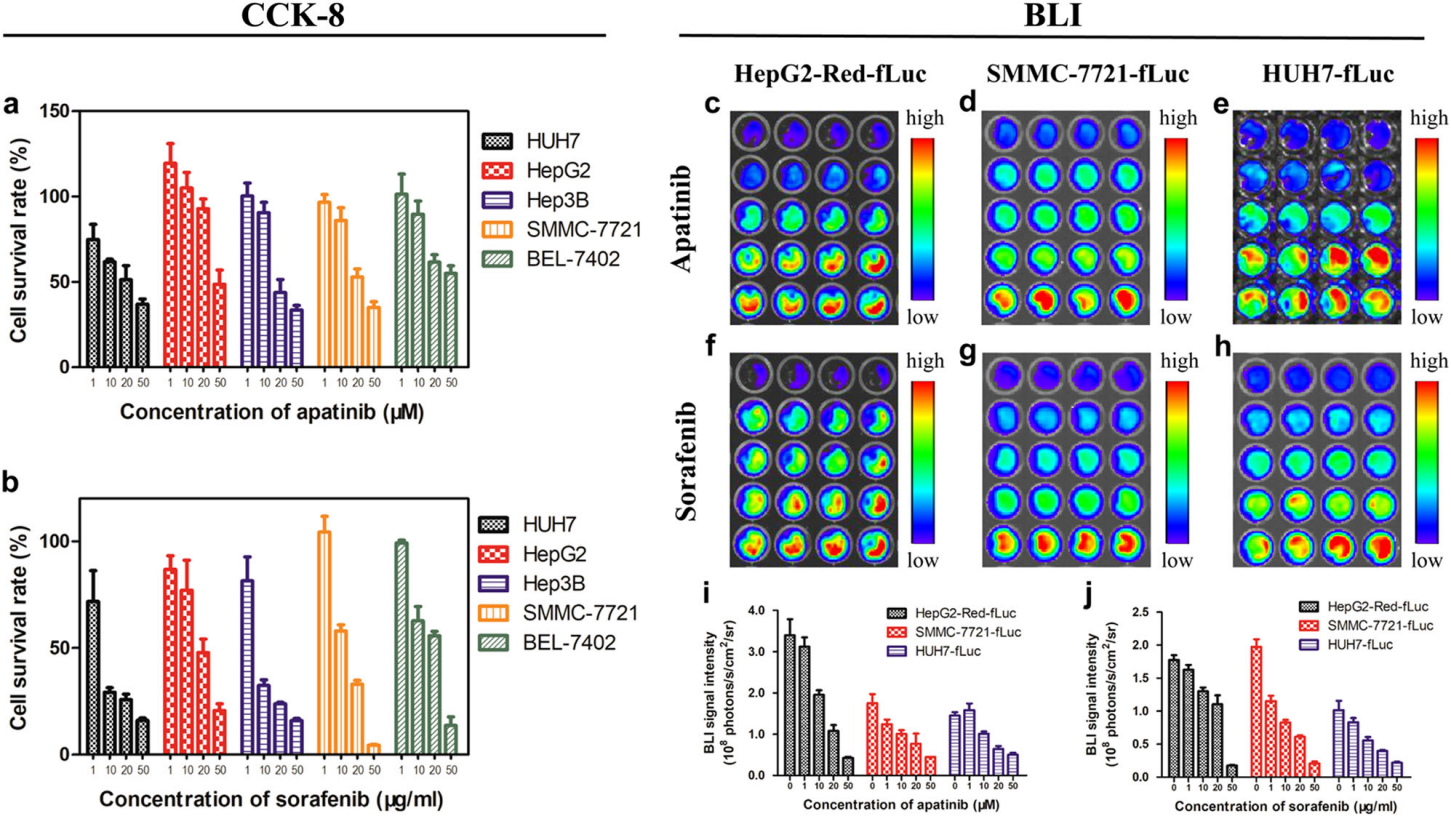
HCC cell viability at different concentrations of apatinib and sorafenib, as evaluated by CCK-8 assays and BLI after 24 h of drug administration. **a** CCK-8 assays of the apatinib and (**b**) sorafenib treatment groups. (**c–h**) BLI images. (**i, j**) Quantification of BLI signal intensity

The cytotoxicity of apatinib and sorafenib to HUH7, HepG2, Hep3B, SMMC-7721, and BEL-7402 human hepatocellular cell lines was evaluated with the CCK-8 assay. The cells were treated with different concentrations of drugs for 24 h. Both apatinib and sorafenib inhibited the proliferation of HCC cells in a dose-dependent manner (Fig. 1a, b). According to the half-maximal inhibitory concentrations of apatinib and sorafenib (Table 1), the latter exerted a more potent inhibitory effect on cell growth.

**Table 1:** Half-maximal inhibitory concentration (IC_50_) of apatinib and sorafenib in hepatocellular carcinoma cell lines

Cell line	IC_50_ of apatinib (μM)	IC_50_ of sorafenib (μM)
HUH7	23.35	5.95
HepG2	49.66	32.25
Hep3B	25.83	8.30
SMMC-7721	28.72	20.08
BEL-7402	54.22	29.17

Expression of the luciferase gene in the HepG2-Red-fLuc, SMMC-7721-fLuc, and HUH7-fLuc cell lines after drug treatment was detected by BLI (Fig. 1c–h). BLI signal intensity, which is proportional to the number of live cells, showed the same inverse dose dependence as cell proliferation, with the signal decreasing as apatinib and sorafenib concentration increased (Fig. 1i, j).

### Antitumor effects of apatinib and sorafenib in subcutaneous and orthotopic HCC models

**Fig. 2:**
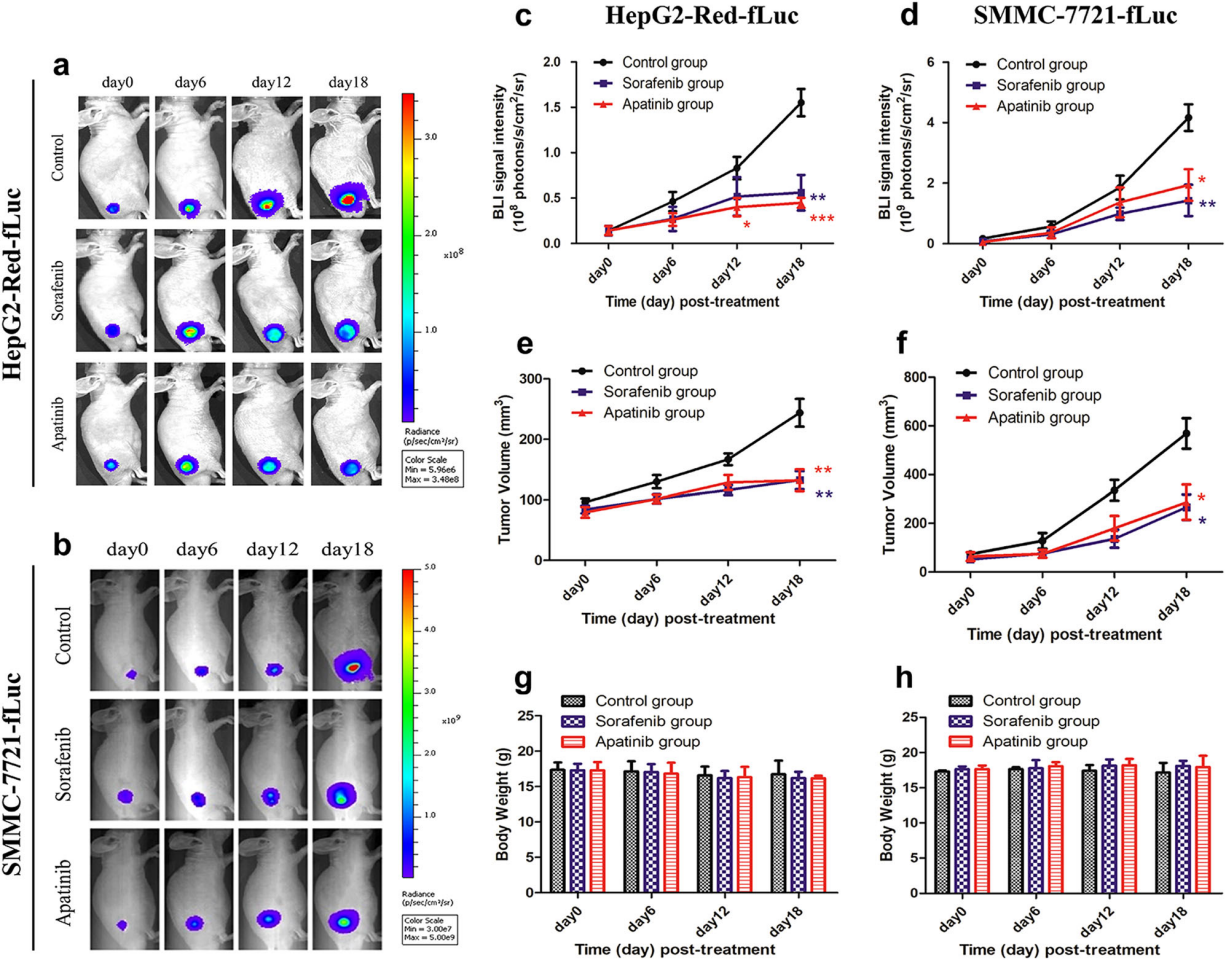
BLI images of subcutaneous liver tumors (*n* = 5 per group). **a** HepG2-Red-fLuc and (**b**) SMMC-7721-fLuc cell-derived tumor-bearing mice on days 0, 6, 12, and 18 post-drug treatment. Changes in BLI signal intensity, tumor volume, and mouse body weight in mice bearing HepG2-Red-fLuc (**c, e, g**) and SMMC-7721-fLuc (**d, f, h**) cell xenograft tumors were evaluated. (*) *P* < 0.05, (**) *P* < 0.01, (***) *P* < 0.001 vs. the control group

We monitored the progression of subcutaneous (HepG2-Red-fLuc and SMMC-7721-fLuc) and orthotopic (HepG2-Red-fLuc) liver tumors in mice treated with apatinib or sorafenib by BLI using BLI signal intensity as a measurement of the antitumor efficacy of the drugs (Fig. 2a–d). The BLI signal of HepG2-Red-fLuc cell-derived tumors was reduced relative to that of the control group starting 12 days after treatment (*P* < 0.05); on day 18, the signal intensities of mice in the apatinib and sorafenib groups were (0.4478 ± 0.05589) × 10^8^ and (0.5603 ± 0.1960) × 10^8^ photons/s/cm^2^/sr, respectively, compared to (1.553 ± 0.1501) × 10^8^ photons/s/cm^2^/sr in control mice. In mice bearing SMMC-7721-fLuc cell-derived tumors, the BLI signal intensities of apatinib-treated and sorafenib-treated mice were (1.939 ± 0.5231) × 10^9^ and (1.429 ± 0.5185) × 10^9^ photons/s/cm^2^/sr, respectively, compared to (4.168 ± 0.4377) × 10^9^ photons/s/cm^2^/sr in the control group on day 18. These results indicate that both apatinib and sorafenib can effectively inhibit HCC growth.

We also measured tumor volumes using digital calipers and found that tumors in the control group were more massive than those in the apatinib and sorafenib treatment groups, with no statistically significant difference between these two groups (Fig. 2e, f). Importantly, there was no difference in body weight among the three groups (Fig. 2g, h), indicating that the drugs are relatively safe and well tolerated.

**Fig. 3:**
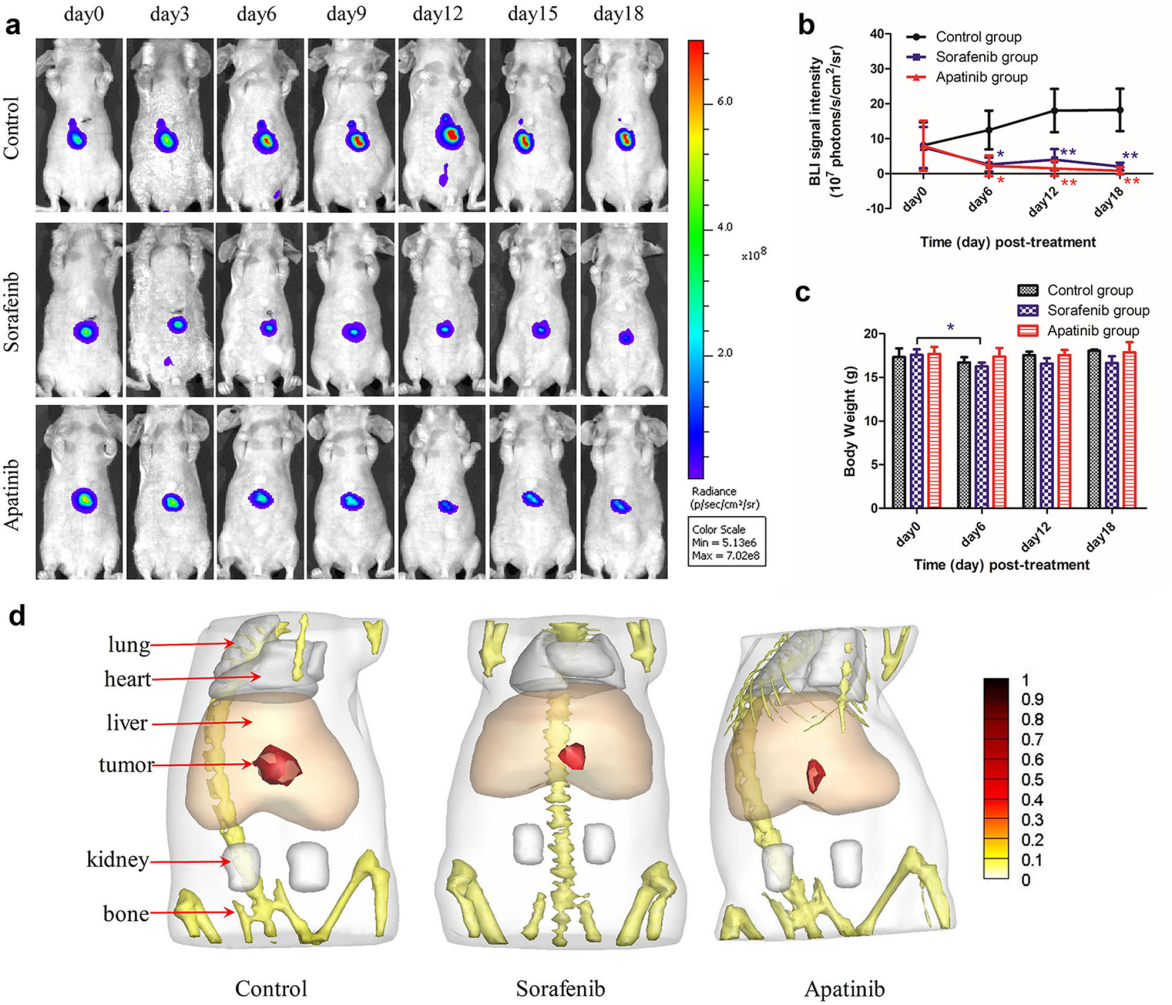
BLI images and 3D BLT reconstruction of orthotopic liver tumors (*n* = 5). **a** BLI signals of orthotopic HepG2-Red-fLuc cell-derived tumor-bearing mice on days 0, 3, 6, 9, 12, 15, and 18 post-drug treatment. **b** BLI signal intensity of mice. (*) *P* < 0.05, (**) *P* < 0.01 vs. the control group. **c** Body weight of mice. (*) *P* < 0.05, day 6 vs. day 0 in the sorafenib group. **d** The 3D BLT reconstruction in the control and sorafenib and apatinib treatment groups on day 20 post-drug treatment

The orthotopic HCC mouse model is a reliable and reproducible tool for investigating tumorigenesis^19,20^. We established this model to simulate the natural tumor microenvironment and monitored orthotopic liver tumor growth during drug treatment by BLI (Fig. 3a, b). Apatinib treatment inhibited tumor growth to a degree similar to sorafenib. In addition, although sorafenib-treated mice showed a statistically significant reduction in body weight on day 6 (16.25 ± 0.2217 g) and a numerical reduction on days 12 (16.58 ± 0.3065 g) and 18 (16.65 ± 0.3884 g) post-treatment compared with day 0 (17.58 ± 0.3198 g), there were no evident changes in apatinib-treated and control mice during treatment (Fig. 3c).

BLI is an imaging modality that is useful for preclinical evaluation of drug efficacy^21^. However, two-dimensional planar BLI provides limited information; detection of a 3D BLI signal can better reveal tumor location and distribution in living animals. Therefore, following drug treatment, we used the sparsity adaptive subspace pursuit reconstruction method^22^ to generate a 3D image of liver tumors based on μCT/BLI signals (Fig. 3d). Tumors were detected in the liver lobes, and the 3D reconstruction confirmed that the tumor volumes were reduced by apatinib and sorafenib treatment, which was consistent with the BLI data.

### FMI and CTA imaging of the antiangiogenic effects of apatinib and sorafenib on orthotopic liver tumors

**Fig. 4:**
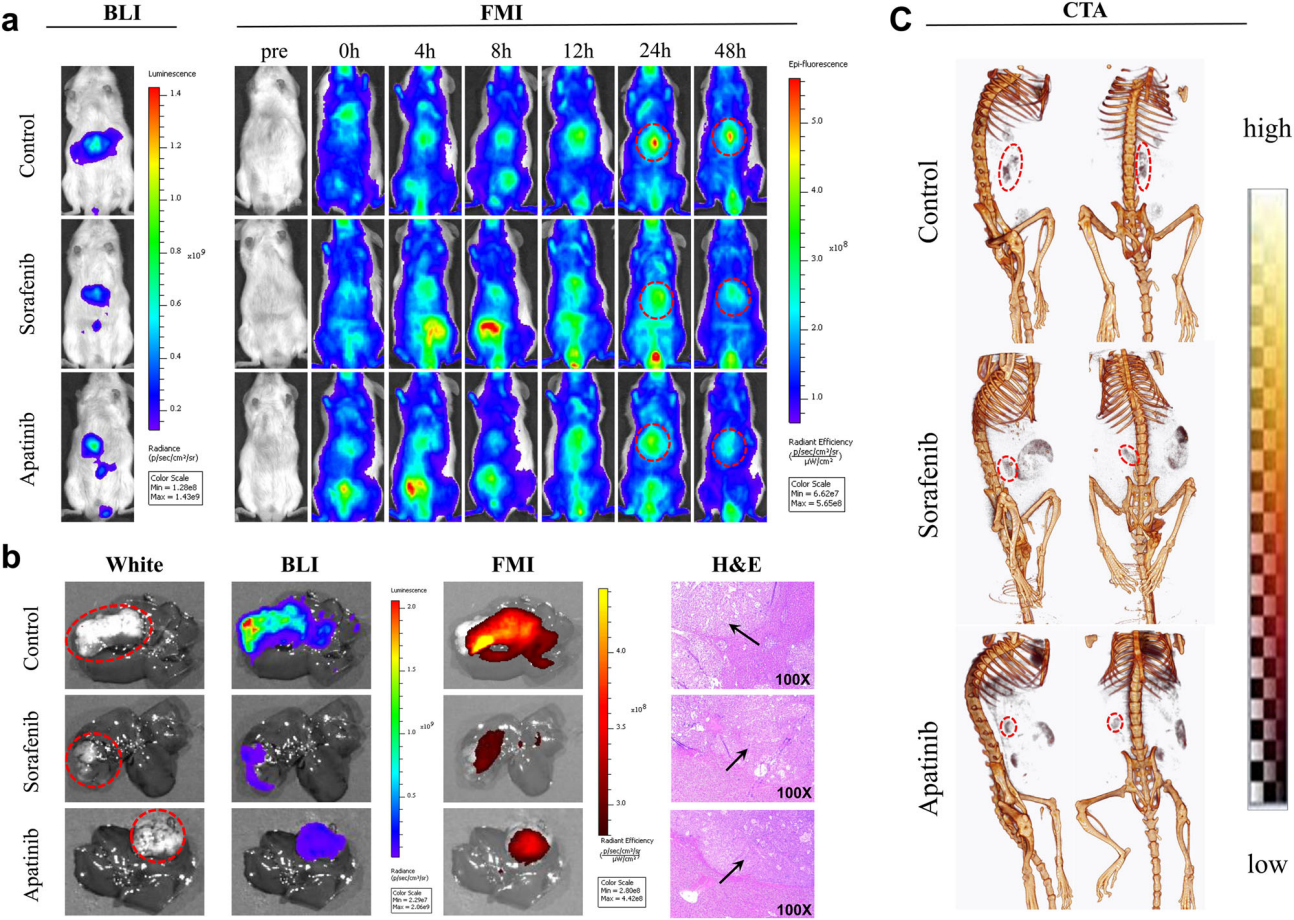
BLI, FMI, and CTA in orthotopic liver tumor-bearing mice on day 20 post-drug treatment (*n* = 3). **a** In vivo BLI and FMI of control and sorafenib and apatinib treatment groups at the time points of pretreatment and 0, 4, 8, 12, 24, and 48 h post-treatment of AngioSense 750 EX. **b** Ex vivo white light imaging, BLI, and FMI livers from mice and H&E staining of liver tumor specimens at 48 h post-treatment with AngioSense 750 EX. Black arrows indicate tumor tissues. **c** The 3D CTA in the control and sorafenib and apatinib treatment groups 15 min after Fenestra VC injection. The red dotted line denotes tumor blood vessel areas

We further used AngioSense 750 EX as a biomarker to assess the antiangiogenic effects of drug treatment. The location of tumors in the liver was first determined by BLI (Fig. 4a), FMI of AngioSense 750 EX was carried out for continuous 48 h observation, and the tumor FMI signal was labeled with red dashed circles. At 24 h post-injection, the signal intensity was lower in the apatinib and sorafenib groups than in the control mice. After in vivo imaging, we dissected the livers with tumors fo*r* ex vivo BLI and FMI (Fig. 4b). The white light and BLI revealed the location of tumors in the liver lobes; AngioSense 750 EX was detected by FMI in the tumor regions. Moreover, the FMI signal was weaker in the apatinib and sorafenib treatment groups than in control tumors, suggesting that angiogenesis was inhibited by both drug treatments.

Unlike optical imaging, the signal detected by CT is independent of imaging depth^3^. We performed CTA of mice with orthotopic liver tumors using the Fenestra VC contrast agent (Fig. 4c) and found that tumors in the apatinib and sorafenib groups had tumors with fewer vessels than those in control mice, as indicated by the red dashed circles. Thus, apatinib behaved similarly as sorafenib to inhibit liver tumor angiogenesis.

### Apatinib inhibits tumor angiogenesis and proliferation but promotes apoptosis of liver tumor cells

**Fig. 5:**
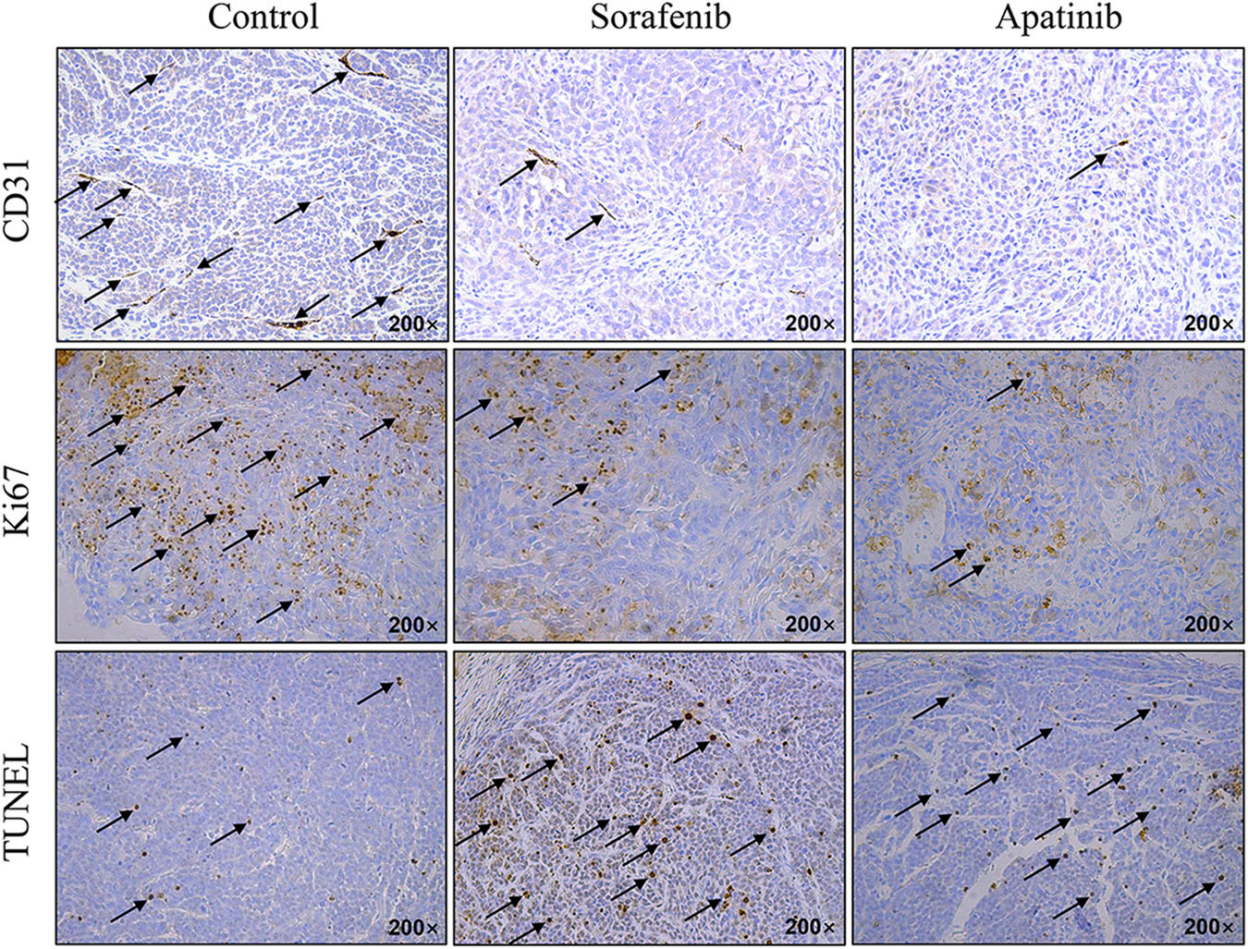
Immunohistochemical analysis of CD31 and Ki67 expression and TUNEL analysis of apoptotic cells in the control and sorafenib and apatinib treatment groups on day 25 post-treatment

To further confirm the antiangiogenic and antiproliferative effects of apatinib and investigate its effects on tumor cell apoptosis, we carried out an immunohistochemical analysis on liver tumor tissues. The data showed that apatinib or sorafenib treatment reduced the expression of the angiogenesis marker CD31 and proliferation marker Ki67 relative to the control group (Fig. 5), and the reduction of CD31 was consistent with the FMI and CTA imaging results. In addition, apatinib and sorafenib treatment increased the number of TUNEL-positive tumor cells compared to the control group. Many apoptotic cells were detected in the apatinib and sorafenib treatment groups, indicating that apatinib and sorafenib not only inhibited liver tumor angiogenesis and tumor cell proliferation but also promoted tumor cell apoptosis to block HCC progression.

### Toxicity of apatinib and sorafenib treatment

**Fig. 6:**
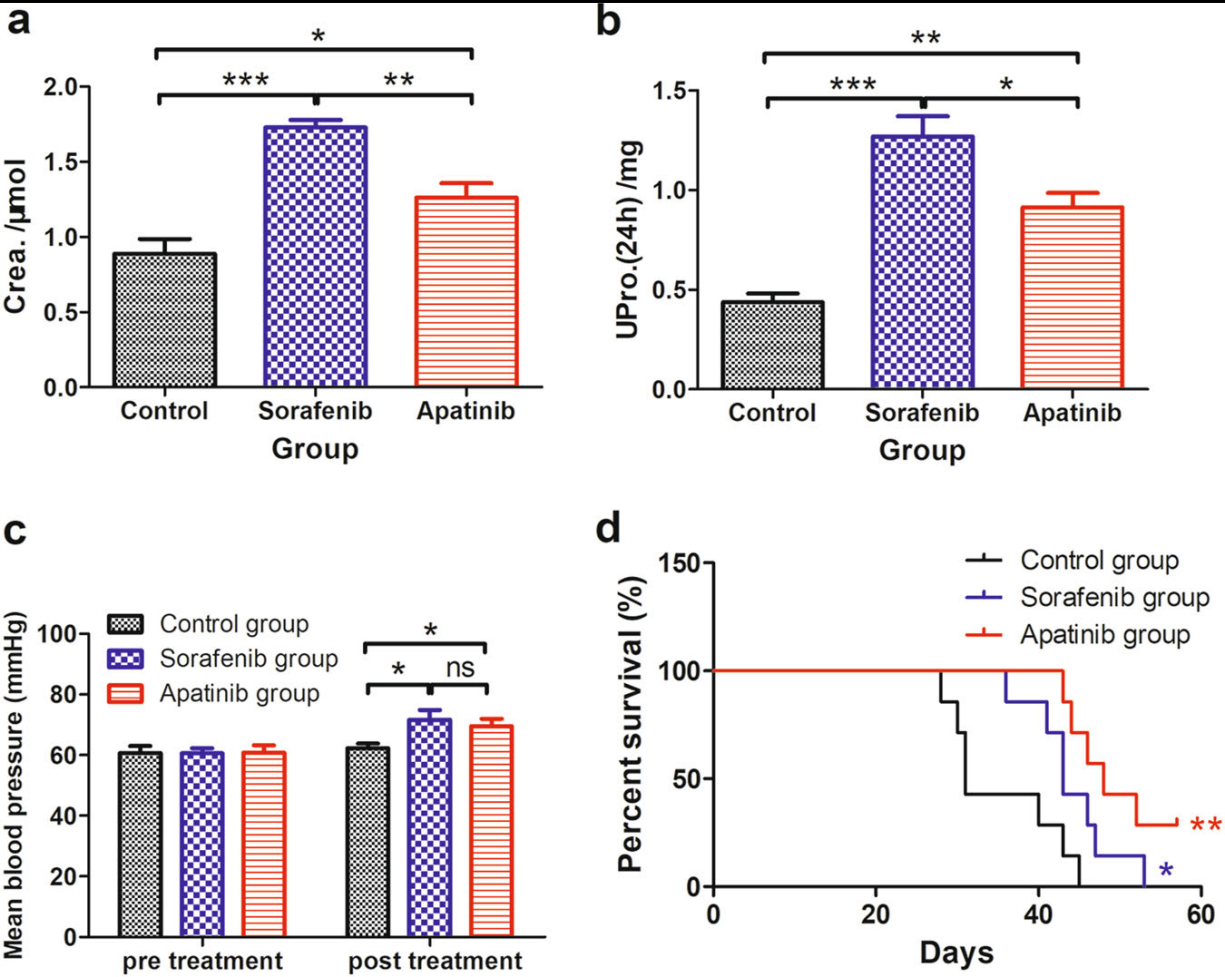
Side effects in the control and sorafenib and apatinib treatment groups. **a** The 24-h urine creatinine (Crea) on day 20 post-treatment (*n* = 4). **b** The 24-h urine protein (UPro) on day 20 post-treatment (*n* = 4). **c** Mean blood pressure pretreatment and post-treatment (day 10) (*n* = 7). **d** Survival rate (*n* = 7). (*) *P* < 0.05, (**) *P* < 0.01, (***) *P* < 0.001, (ns) non-significant, *P* ≥ 0.05

We next evaluated the side effects of apatinib and sorafenib by measuring urine creatinine and protein levels and blood pressure in tumor-bearing mice under different treatments. Creatinine concentration in urine was higher in mice treated with apatinib (1.261 ± 0.09605 μmol) and sorafenib (1.727 ± 0.05054 μmol) relative to control animals (0.8883 ± 0.09812 μmol) (Fig. 6a), whereas urine protein levels were more than twice as high in the apatinib (0.9136 ± 0.07150 mg) and sorafenib (1.269 ± 0.1018 mg) groups compared to the control group (0.4375 ± 0.04323 mg) (Fig. 6b). These results suggest that apatinib or sorafenib treatment can lead to kidney dysfunction and that sorafenib causes more severe kidney damage than apatinib, which is an essential consideration for clinical application.

Serum ALT and AST levels reflect the degree of liver damage^23^. We found that the levels of both enzymes were higher in the apatinib and sorafenib treatment groups than in control mice (Table 2), indicating that the drugs caused some degree of liver damage. H&E staining of major organs showed no visible tissue damage in the heart, spleen, lung, or kidney caused by drug treatment. However, some damage was observed in the livers of apatinib-treated and sorafenib-treated mice, but this was more severe in the latter group than in the former group (Fig. S3).

**Table 2:** Serum alanine transaminase (ALT) and aspartate transaminase (AST) levels in the control, sorafenib, and apatinib groups (*n* = 4)

Group	ALT (IU/l)	AST (IU/l)
Control	47.75 ± 1.11	152.5 ± 11.81
Sorafenib	82.83 ± 19.25	210.3 ± 21.70
Apatinib	67.00 ± 7.10	200.7 ± 21.53

We also compared the mean blood pressure in mice before and after drug treatment. There was no significant difference in the mean blood pressures between control (60.74 ± 2.299 mmHg) mice and those treated with apatinib (60.91 ± 2.397 mmHg) and sorafenib (60.64 ± 1.642 mmHg) prior to drug administration (Fig. 6c). We then measured blood pressure on days 5, 10, and 15 post-treatment and found that on day 10, the mean blood pressure was elevated in apatinib-treated and sorafenib-treated mice relative to controls, which showed no significant change (69.53 ± 2.538 and 71.71 ± 3.257 mmHg, respectively vs. 62.31 ± 1.518 mmHg).

In addition to the abovementioned drug-induced side effects, rashes and diarrhea were observed in mice treated with sorafenib but not in those treated with apatinib (Fig. S4). The median survival time in the apatinib and sorafenib treatment groups was 48 and 43 days, respectively, compared to 31 days in control mice in tumor xenograft models (Fig. 6d). Thus, apatinib treatment can prolong survival in HCC model mice relative to vehicle or sorafenib treatment.

## Discussion

In this study, we assessed the therapeutic efficacy of apatinib and sorafenib in HCC by multimodality molecular imaging. BLI, BLT, FMI, and CTA were fully utilized to sensitively and dynamically assess the antitumorigenic and antiangiogenic effects of drug treatment and to examine the side effects. Our results demonstrated that apatinib inhibited HCC growth and angiogenesis both in vitro and in vivo with efficacy that was comparable to that of sorafenib but with fewer side effects. In general, our preclinical assessment of apatinib demonstrates that it has clinical application potential for the treatment of HCC in patients.

We used multimodal molecular imaging to evaluate the therapeutic response, with appropriate adjustments made in the experimental protocol. This approach is critical in the evaluation of antitumor drugs. BLI is a more sensitive and effective method for detecting cancer lesions and evaluating drug treatment efficacy than tumor volume measurement since it provides quantitative information on living tumor cells before and after treatment. In this study, the inhibition of HCC cell proliferation by apatinib was detected in HepG2-Red-fLuc subcutaneous tumor-bearing mice at 12 days post-treatment by BLI, whereas the difference relative to the control mice was only observed on day 18 by measuring tumor volume. Moreover, the latter is not feasible for orthotopic HCC models at early stages, whereas the inhibitory effects on tumor growth utilizing BLI are detectable as early as 6 days post-treatment. While BLI can only provide 2D information on orthotopic HCC tumors, BLT can reveal details of both tumor growth and structure. In the present study, the 3D bioluminescence distribution of orthotopic liver tumors was reconstructed to evaluate drug treatment efficacy, which provided both the tumor location and volume information at the molecular and cellular levels.

Angiogenesis plays a vital role in liver tumor growth^9^ and is known to be inhibited by both apatinib and sorafenib^10,11^. We carried out FMI using the AngioSense 750 EX fluorescence imaging contrast agent; however, although this probe can provide sensitive and specific imaging information, the fluorescence signal cannot be detected in deep tissues. Since the CT signal is independent of imaging depth, we performed 3D CTA of orthotopic liver tumors using the Fenestra VC imaging contrast agent. The in vivo FMI and CTA imaging results revealed that apatinib and sorafenib suppressed angiogenesis relative to vehicle-treated control animals. Immunohistochemical analysis of CD31 and Ki67 and TUNEL positivity confirmed that tumor angiogenesis and tumor cell proliferation were inhibited, whereas apoptosis was enhanced by drug treatment.

The side effects of antitumor drugs are a critical clinical consideration. In this study, we monitored changes in urine creatinine and protein levels, serum ALT and AST levels, and blood pressure to assess the toxicity of apatinib and sorafenib. The results showed that both drugs caused some degree of liver and kidney dysfunction and increased blood pressure, although apatinib did not cause other side effects, such as diarrhea, rashes, and weight loss, that were associated with sorafenib treatment. Thus, apatinib may be a relatively safer alternative for HCC treatment than sorafenib.

In conclusion, our results demonstrated that multimodality molecular imaging is a more sensitive and functional method for evaluating the therapeutic effects of apatinib on HCC, as it revealed early aspects of the drug treatment response. Apatinib has antitumorigenic and antiangiogenic effects in HCC both in vitro and in vivo that are comparable to those of sorafenib but has fewer toxic side effects. Our findings provide preclinical evidence for further investigations on the clinical applicability of apatinib to HCC patients.

## Supplementary information

Supplementary Information
