# Supplementary material for: Antitumorigenic and antiangiogenic efficacy of apatinib in liver cancer evaluated by multimodality molecular imaging
Source: Exp Mol Med. 2019 Jul 8;51(7):76. doi: 10.1038/s12276-019-0274-7 (PMC6802662; doi:10.1038/s12276-019-0274-7)
Supplement: Supplementary file 1 — Supplementary Information [file 12276_2019_274_MOESM1_ESM.docx]

Research article

**Antitumorigenic and antiangiogenic efficacy of apatinib in liver cancer evaluated by multimodality molecular imaging**

Qian Liang^1,2,3,#^, Lingxin Kong^1,2,3,#^, Yang Du^1,2,3,*^, Xu Zhu^4,*^, Jie Tian^1,2,3,5,6,*^

^1^. CAS Key Laboratory of Molecular Imaging, the State Key Laboratory of Management and Control for Complex Systems, Institute of Automation, Chinese Academy of Sciences, Beijing, 100190, China

^2^. University of Chinese Academy of Sciences, Beijing, 100080, China

^3^. Beijing Key Laboratory of Molecular Imaging, Beijing, 100190, China

^4^. Key Laboratory of Carcinogenesis and Translational Research (Ministry of Education/Beijing), Department of Interventional Therapy, Peking University School of Oncology, No. 52 Fucheng Road, Haidian District, Beijing 100142, China

^5^. Beijing Advanced Innovation Center for Big Data-Based Precision Medicine, School of Medicine, Beihang University, Beijing, 100191, China

^6^. Engineering Research Center of Molecular and Neuro Imaging of Ministry of Education, School of Life Science and Technology, Xidian University, Xi’an, Shaanxi, 710126, China

^#^ Qian Liang and Lingxin Kong contributed equally to this work

* To whom correspondence should be addressed: Yang Du, E-mail: [yang.du@ia.ac.cn](mailto:yang.du@ia.ac.cn). Phone: +86 10 62611658, Fax: +86 10 62611658; Xu Zhu, E-mail: drzhuxu@163.com. Phone: +86 10 88196001, Fax: +86 10 88196001; Jie Tian, E-mail: jie.tian@ia.ac.cn. Phone: +86 10 82618465, Fax: +86 10 62527995.

**Running title:** Apatinib efficacy in HCC evaluated by multimodal imaging

**Supplementary Information**

**Reconstruction methods**

BLT reconstruction procedure of the orthotopic liver tumor model is shown in Fig. S4. Firstly, we acquired μCT data and BLI data through μCT/BLI system. Secondly, the main organs, including muscles, heart, lung, liver, kidney, and bone, were segmented to establish the heterogeneous mouse model with region growing and threshold segmentation methods. The assembled mouse body was discretized into surface and volumetric mesh data by finite element method. And then, the bioluminescence signals were mapped onto the volumetric mesh surface in terms of spatial location and energy distribution. Finally, photo propagation model was built to reconstruct 3D BLT signal distribution in the mouse using reconstruction algorithm.

Sparsity adaptive subspace pursuit (SASP) method ^1^ was utilized to reconstruct the 3D bioluminescence distribution of tumors in different groups. The subspace projection and correlation maximization approach, bottom-up sparsity adaptive strategy, and backtracking technique were adopted to guarantee the accurate, efficient, and robust BLT reconstruction results. The main steps of SASP algorithm are as follows:

Firstly, we apply matched filtering to residual vector $r^{n-1}$ to get the residual correlations vector $c^{n-1}$in the n-th iteration.

$c^{n-1}=A^{T}r^{n-1}$ (1)

Secondly, the measurements vector $\Phi$ is projected onto the subspace spanned by the columns of the matrix$A_{J^{n}}$.

$\Phi_{p,J^{n}}=proj(\Phi,A_{J^{n}})=A_{J^{n}}A_{J^{n}}^{+}\Phi$ (2)

The coefficients vector of the subspace projection is$x_{p}$.

$x_{p}=A_{J^{n}}^{+}\Phi$ (3)

Thirdly, the $K$ indices with the largest correlation magnitudes of $x_{p}$ are selected to build a new support set$I$. The vector $\Phi$ is projected onto the subspace spanned by$A_{I}$.

$\Phi_{p,I}=proj(\Phi,A_{I})=A_{I}A_{I}^{+}\Phi$ (4)

Fourthly, compute the new residual vector:

$r=resid(\Phi,A_{I})=\Phi-proj(\Phi,A_{I})=\Phi-A_{I}A_{I}^{+}\Phi$ (5)

Finally, determine whether to terminate the iteration and update the unknown bioluminescent yields:

$x=\{\hat{x}|\text{ }\hat{x}_{I^{n}}=A_{I^{n}}^{+}\Phi\text{ and }\hat{x}_{\{1,2,3...,N\}-I^{n}}=0\}$ (6)

**
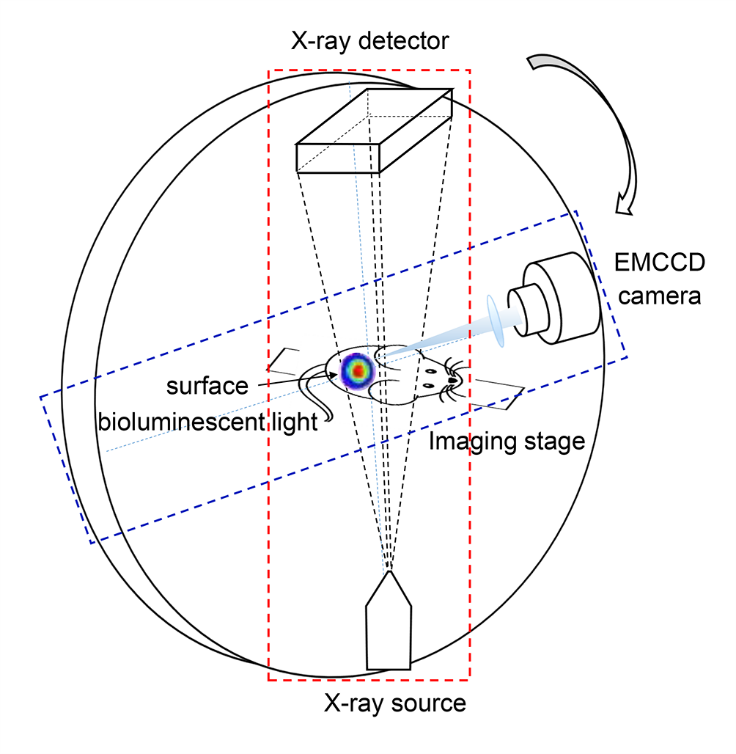
**

**Figure S1.** Schematic illustration of μCT/BLI imaging system. Different colors represent different imaging modalities. Red and blue dotted lines show the μCT and BLI systems, respectively.


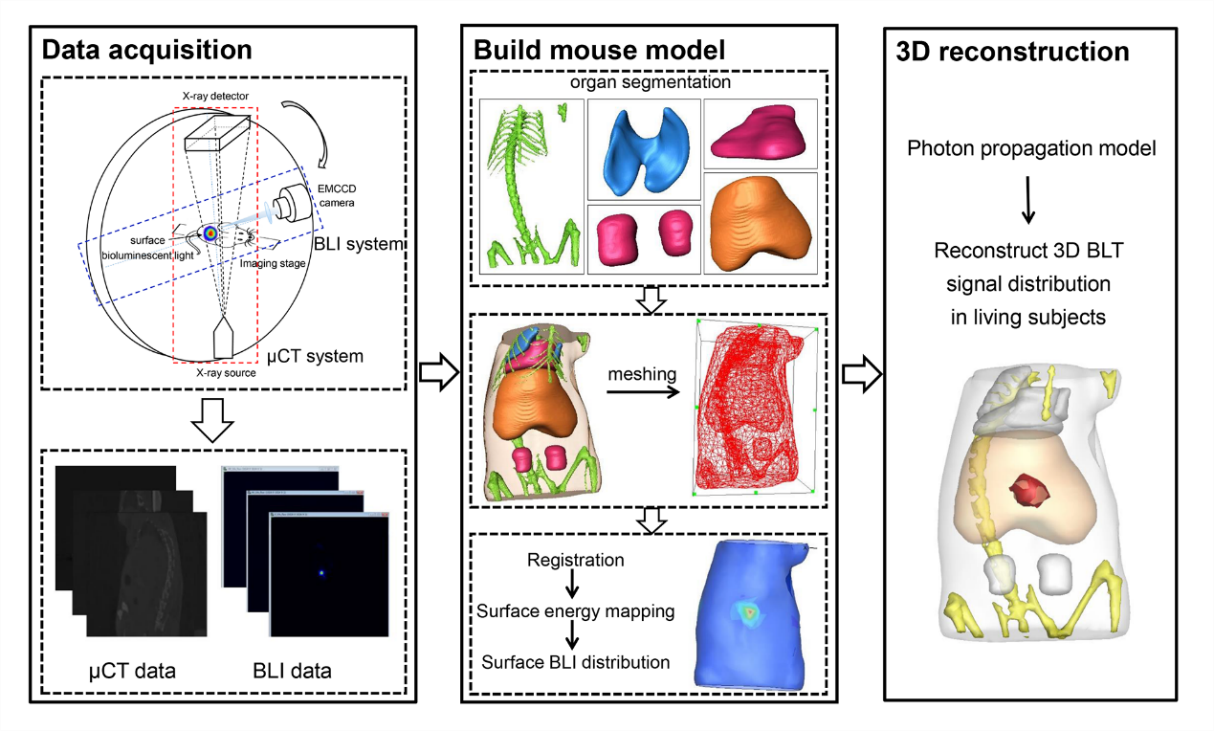


**Figure S2.** 3D BLT reconstruction procedure of the orthotopic liver tumor model.


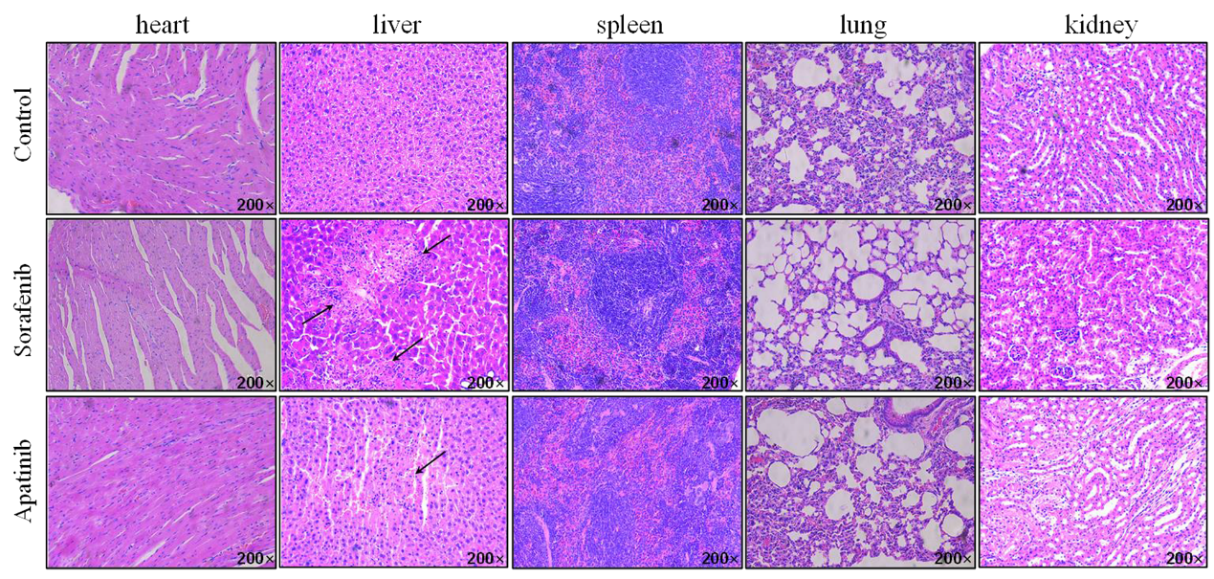


**Figure S3.** H&E staining (200×) of heart, liver, spleen, lung, and kidney tissue specimens from mice in the control and sorafenib and apatinib treatment groups.


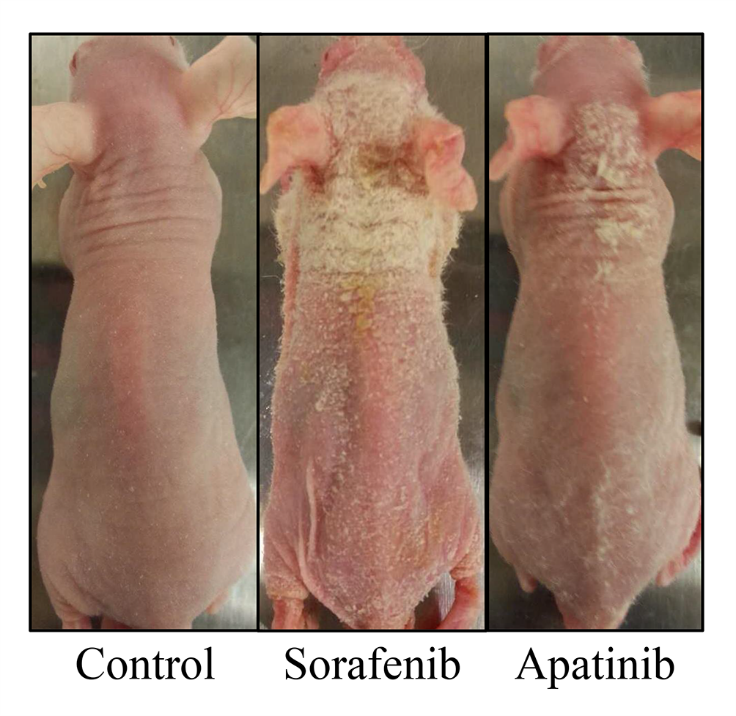


**Figure S4.** Skin rashes of mice in the control and sorafenib and apatinib treatment groups.

**References**

1 Ye, J. *et al.* Fast and robust reconstruction for fluorescence molecular tomography via a sparsity adaptive subspace pursuit method. *Biomed. Opt. Express* **5**, 387-406 (2014).
